# Blockchain in Health Information Systems: A Systematic Review

**DOI:** 10.3390/ijerph21111512

**Published:** 2024-11-14

**Authors:** Aleika Lwiza Alves Fonsêca, Ingridy Marina Pierre Barbalho, Felipe Fernandes, Ernano Arrais Júnior, Danilo Alves Pinto Nagem, Pablo Holanda Cardoso, Nícolas Vinícius Rodrigues Veras, Fernando Lucas de Oliveira Farias, Ana Raquel Lindquist, João Paulo Q. dos Santos, Antonio Higor Freire de Morais, Jorge Henriques, Marcia Lucena, Ricardo Alexsandro de Medeiros Valentim

**Affiliations:** 1 Laboratory of Technological Innovation in Health (LAIS), Federal University of Rio Grande do Norte (UFRN), Natal 59010-090, Brazil; ingridy.marina@lais.huol.ufrn.br (I.M.P.B.); felipe.ricardo@lais.huol.ufrn.br (F.F.); ernano.arrais@lais.huol.ufrn.br (E.A.J.); danilo.nagem@lais.huol.ufrn.br (D.A.P.N.); pablo.holanda@lais.huol.ufrn.br (P.H.C.); nicolas.vinicius@lais.huol.ufrn.br (N.V.R.V.); fernando.farias@lais.huol.ufrn.br (F.L.d.O.F.); raquel.lindquist@lais.huol.ufrn.br (A.R.L.); ricardo.valentim@lais.huol.ufrn.br (R.A.d.M.V.); 2 Laboratory of Intervention and Analysis of Movement, Department of Physical Therapy, Federal University of Rio Grande do Norte (UFRN), Natal 59000-000, Brazil; 3 Advanced Nucleus of Technological Innovation (NAVI), Federal Institute of Rio Grande do Norte, Natal 59015-000, Brazil; joao.queiroz@lais.huol.ufrn.br (J.P.Q.d.S.); higor.morais@lais.huol.ufrn.br (A.H.F.d.M.); 4 Department of Informatics Engineering, Center for Informatics and Systems of the University of Coimbra, Universidade de Coimbra, 3030-788 Coimbra, Portugal; jh@dei.uc.pt; 5 Department of Informatics and Applied Mathematics (DIMAP), Federal University of Rio Grande do Norte (UFRN), Natal 59078-900, Brazil; marciaj@dimap.ufrn.br

**Keywords:** blockchain, health information systems, public health, electronic patient record, health system

## Abstract

(1) Background: With the increasing digitalization of healthcare systems, data security and privacy have become crucial issues. In parallel, blockchain technology has gradually proven to be an innovative solution to address this challenge, as its ability to provide an immutable and secure record of transactions offers significant promise for healthcare information management. This systematic review aims to explore the applications of blockchain in health information systems, highlighting its advantages and challenges. (2) Methods: The publications chosen to compose this review were collected from six databases, resulting in the initial identification of 4864 studies. Of these, 73 were selected for in-depth analysis. (3) Results: The main results show that blockchain has been used mainly in electronic health records (63%). Furthermore, it was used in the Internet of Medical Things (8.2%) and for data sharing during the COVID-19 pandemic (6.8%). As advantages, greater security, privacy, and data integrity were identified, while the challenges point to the need for standardization and regulatory issues. (4) Conclusions: Despite the difficulties encountered, blockchain has significant potential to improve healthcare data management. However, more research and continued collaboration between those involved are needed to maximize its benefits.

## 1. Introduction

The rise of blockchain technology in recent years is largely due to its decentralized data storage and transfer characteristics, where there is no need for a central authority controlling the content that is added to the network. In addition, this technology can potentially solve problems related to privacy, security, and data integrity [1]. Since 2008, when it was introduced by the cryptocurrency Bitcoin as a mechanism used to record currency transactions, blockchain technology has been attracting a lot of attention in various areas that already see the possibilities for progress with the incorporation of this technology into their sectors, extending beyond applications for financial use [2,3,4].

The main feature of blockchain technology is its growing collection of data, grouped in blocks, in a decentralized, secure, and immutable way [3,5]. A block can be understood as a collection of records. Each block is linked to its previous and subsequent blocks in the form of a chain, hence the term “blockchain”. This is achieved using a cryptographic technique called hash, which is nothing more than a security key or fingerprint. The hash is generated from a function that receives a variable input value and returns a fixed-size hash as output [6]. This strategy enables auditing, tracking, and integrity of stored data.

The health sector is a field that makes intensive use of sensitive data from users of various health services and therefore commonly has to deal with factors such as security, confidentiality, and interoperability [7,8,9]. This includes data access operations such as sharing, storing, and exchanging information [10]. Electronic health records (EHRs) play an important role in the digital health transition, acting in real-time communication, storage, and query-oriented processing [11,12,13]. Current electronic health record systems face challenges related to interoperability and security, which is why data security on these platforms is a significant research topic [14,15,16]. Studies to date based on blockchain for electronic health records have adopted various methods to improve security and privacy, especially when sharing data [11,17,18].

Blockchain technology, given its distinctive characteristics, is emerging as a promising solution to the challenges mentioned above [19,20]. The ability to preserve data is one of the main objectives of using this technology, particularly in the health sector, which is subject to massive sharing and dissemination of a significant amount of data [21,22,23]. To maintain users’ privacy in healthcare services and to exchange data with other institutions in the health ecosystem, access control, data integrity, and interoperability are crucial [12,19,24,25].

In light of the growing potential of blockchain technology in the context of health information systems around the world and its progress in recent years, it is important to evaluate the most recent scenarios of its use, understand its use considering its progressive nature, and explore the prospects for the use of blockchain [2,3,15,26,27,28,29]. As such, this review aims to explore the literature to identify the limitations and challenges faced by researchers and professionals in implementing this technology as well as explaining how this technology has been used and its trends. The results of this review can be used as a reference for the development of new studies.

## 2. Materials and Methods

The systematic review presented was based on the systematic review guidelines proposed by Kitchenham (2004) [30] and conducted based on the Preferred Reporting Items for Systematic Reviews and Meta-Analyses (PRISMA) checklist [31]. Furthermore, it was registered in the International prospective register of systematic reviews (PROSPERO) under registration No. CRD42024564961 [32]. To guide the search strategy for relevant studies, the following research questions were defined and included in the review (Table 1):

The process of identifying primary studies related to the object of investigation of this systematic review initially consisted of searches in the repositories. Before executing the review protocol, we conducted searches across multiple databases to gain insight into feedback in advance and identify the most appropriate databases. Thus, the selected repositories returned publications on technology and/or health from journals with a high impact factor and worldwide recognition. They are ACM Digital Library, Web of Science (WOS), ScienceDirect (Elsevier), IEEE Explore, PubMed, and Springer. The searches in the databases were carried out on 31 October 2023.

Similar to the repository selection, we performed searches with various combinations of keywords defined in the research protocol to define the best string that would return the most representative articles to achieve the research objective. The search string (SS01) used was assembled with the keywords related to the topic and some of their synonyms, resulting in the following:

SS01: (“blockchain”) AND (“health” OR “digital health” OR “health system” OR “medical” OR “health system information” OR “electronic medical record”).

After identifying and defining the initial set of records, screening was performed to select a subset of eligible primary studies. This process was organized and executed applying three elementary procedures: (i) Inclusion Criteria—IC; (ii) Exclusion Criteria—EC; and (iii) Quality Assessment Criteria—QA.

In procedure (I), a subset of primary studies was selected based on the Inclusion Criteria (Table 2), which were applied using the filters available in the repositories.

In procedure (II), screening was carried out based on the Exclusion Criteria (Table 3), based on reading the titles, abstracts, and keywords of the set of primary studies. The Ryyan tool [33], a web application for systematic reviews, helped in carrying out step (II).

The article screening process was based on the Quality Assessment Criteria (QA) (Table 4). To identify the answers to the RQs (Table 1), a spreadsheet was created to provide guidance on the main points expected in each study. Following this approach, it was possible to carry out a reading directed at the previously selected key points.

An evaluation metric, called score, was used to evaluate and classify the studies, as demonstrated in Equation (1). The score is calculated as the average of the weights (*w*) assigned to each QA criterion. The weight (*w*), which can vary between 0, 0.5, and 1.0, measures how satisfactory the response of that article is to a specific QA criterion, as shown in Equation (2). Primary articles that scored 0.5 or higher (i.e., 0.5 ≤score≤ 1.0) were considered eligible for this systematic review. Only one reviewer assigned scores and the elementary data from the final set of eligible studies, extracted based on the research questions.

(1) $$\frac{1}{n_{QA}}\sum_{i=1}^{n_{QA}}w_{QA_{i}}\;$$

- $n_{QA}$: variable used to represent the total value of Quality Assessment Criteria;
- $w_{QA}$: variable used to determine the value referring to the weight *w* assigned to the Quality Assessment Criteria under analysis (see the possible values in Equation (2)).

(2) $$w_{QA}=\left\{\begin{array}{cc} 1.0, & \mathrm{yes},\;\mathrm{completely}\;\mathrm{describes}\\ 0.5, & \mathrm{yes},\;\mathrm{partially}\;\mathrm{describes}\\ 0, & \mathrm{does}\;\mathrm{not}\;\mathrm{describe} \end{array}\right.$$

**Figure 1:**
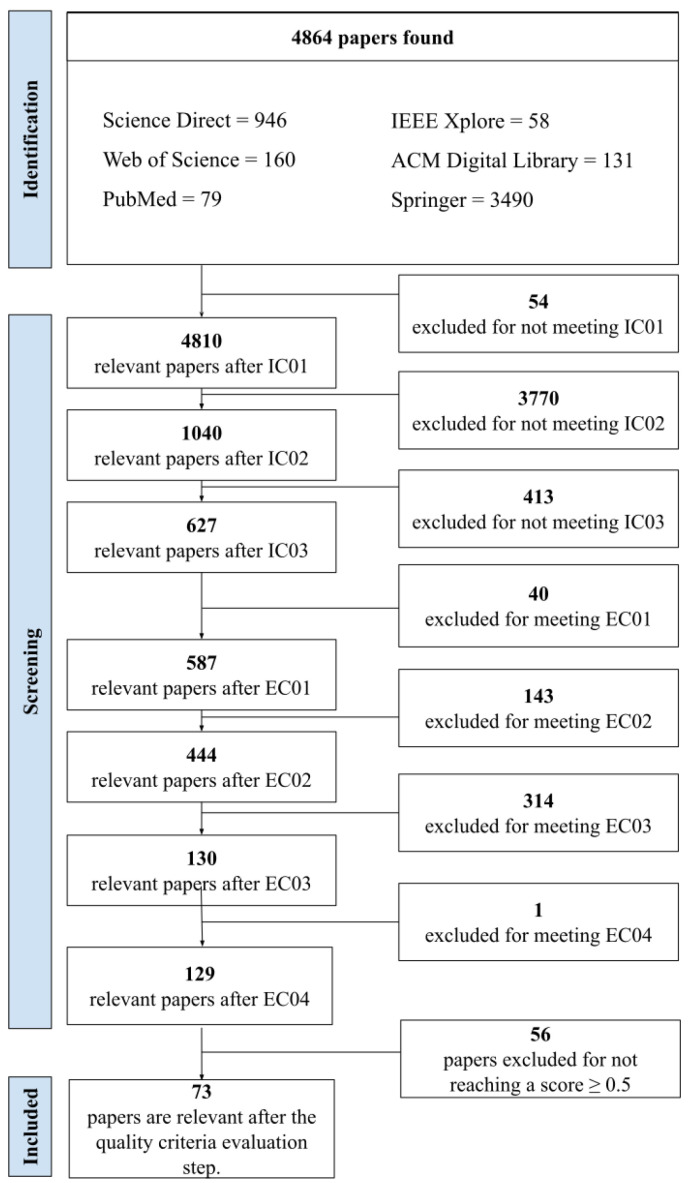
Execution of the study selection protocol.

Figure 1 shows the details of how this systematic review was carried out, highlighting the number of articles included and excluded at each stage.

## 3. Results

The quantitative results of the execution of the systematic review protocol are presented in Figure 1. After applying the search string to the selected databases (procedure I), 4864 studies were identified. This set of studies was refined by carrying out procedure II, through which 4277 studies were excluded for not meeting the Inclusion Criteria (Table 2), and 458 were removed for meeting the Exclusion Criteria (Table 3). Subsequently, the remaining 129 studies were analyzed according to the Quality Assessment Criteria (Table 4). After reading and scoring each one, 56 studies were excluded because they did not meet the score defined in the protocol. Lastly (procedure III), a set of 73 studies were classified as eligible and included in this systematic review, with the aim of answering the research questions (Table 1). The 73 included studies were added to the Supplementary Materials in tabular form in Table S1. The results will be presented according to the sequence of the research questions presented and, based on the completion of Table S1, used as an analysis strategy for the included studies. In addition to being used as a basis for scoring the Evaluation Criteria, this table was also used to classify the studies and collect the most relevant information, according to what the research questions wanted to answer.

### 3.1. Rq01—Which Health Information Systems Are Using Blockchain Technology?

**Figure 2:**
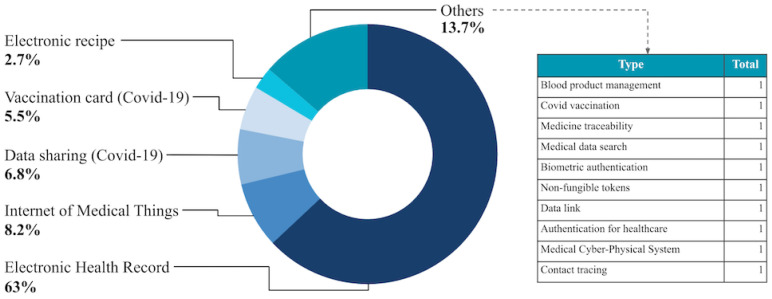
Distribution of blockchain implementation in health information systems.

Based on the studies included, and as can be seen in Figure 2, blockchain technology has been used in the context of health information system applications in scenarios such as electronic health records, Internet of Medical Things, data sharing in the fight against COVID-19, COVID-19 digital health passports, and e-prescriptions, among others, which had only one occurrence and are also listed in Figure 2.

The use of blockchain in solutions for electronic health records was highlighted in 63% of the studies analyzed, covering a wide range of solution initiatives in the context of health information systems [11,14,16,17,34,35,36,37,38,39,40,41,42,43,44,45,46,47,48,49,50,51,52,53,54,55,56,57,58,59,60,61,62,63,64,65,66,67,68,69,70,71,72,73,74,75]. This approach includes managing a large amount of medical information about patients, such as medical history, test results, prescriptions, and health records, which provides greater security and data integrity. Even with the different frequencies of electronic health records, other approaches to utilizing blockchain have also been found. Studies on the Internet of Medical Things (IoMT) describe the deployment of blockchain to establish secure shared sessions between authenticated devices, preventing unauthorized access [64,76,77,78,79,80,81,82]. Another issue is that during the COVID-19 pandemic, blockchain was also implemented to promote the secure sharing of data related to the pandemic and to help issue vaccination certificates and essential documents during this period [83,84,85,86,87,88,89,90,91,92,93]. In addition, although less common, the use of blockchain for electronic prescriptions is also worth highlighting, showing its potential to innovate and protect various areas of health information systems [94,95]. Other more isolated themes were encrypted medical data [96], biometric authentication [97], tokenization for health information [98], blockchain native data linkage [99], distributed authentication systems [100], and medical cyber–physical systems [101].

### 3.2. Rq02—What Are the Most Widely Used Practices and Models for Implementing Blockchain in Health Information Systems?

To answer RQ02 on the practices and models most used in the implementation of the blockchain in the selected primary studies, we sought to classify them as Ethereum and Hyperledger Fabric, as well as Hyperledger Aries and Hyperledger Indy, which are tools within Hyperledger.

Ethereum is a public blockchain that allows you to create and execute automatic programs called smart contracts, as well as decentralized applications. Any member of the Ethereum network can create new smart contracts and customize the blockchain’s functionalities without the approval of other network members. Hyperledger Fabric, on the other hand, is a private blockchain with permission, offering more control over who can participate and access the network [46].

Hyperledger Aries provides the tools for the secure exchange of information and digital identities, helping to authenticate and verify data. Hyperledger Indy provides a decentralized structure specialized in providing identity management, so that users have complete autonomy over their information and decide who has access to which part of their data. Both Aries and Indy are geared towards digital identities, with Indy providing the database and Aries facilitating the exchange of information [94].

**Figure 3:**
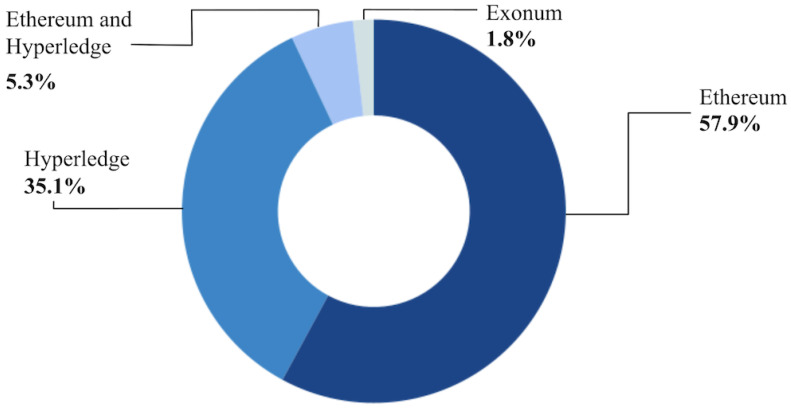
Blockchain models used in the studies.

Of the 73 articles included, only 57 gave more details about the blockchain structure used to develop the proposed solution. As Figure 3 shows, 33 (57.9%) used Ethereum, 20 (35.1%) used some version of Hyperledger (Hyperledger Fabric, Hyperledger Aries, and Hyperledger Indy), with Hyperledger Fabric being the most used in 18 of the cases listed. Only three (5.3%) of the papers still used both Ethereum and Hyperledger in their solutions [90,94,95]. In addition to the main platforms mentioned above, one of the papers used Exonum, an open-source blockchain structure [69].

Table 5 below shows the relationship between the models used and the applications in which they are most commonly used.

Also related to the practices used to implement the blockchain, a classification was made according to the permissiveness of the network, i.e., who can participate in the validation of transactions and the visibility of data stored on the blockchain. To this end, the studies were categorized into the following [102]:

- Public: Any member can join the blockchain system and participate in the consensus protocol. Any interested party can join the network, obtain a complete copy of the ledger, and autonomously validate the transactions it contains.
- Private: The system is centralized from a governance point of view and requires all participants to trust the entity that manages it.
- Consortium: A consortium blockchain can be used to resolve trust issues between consortium members, but parties outside the consortium still need to trust the consortium itself.

**Figure 4:**
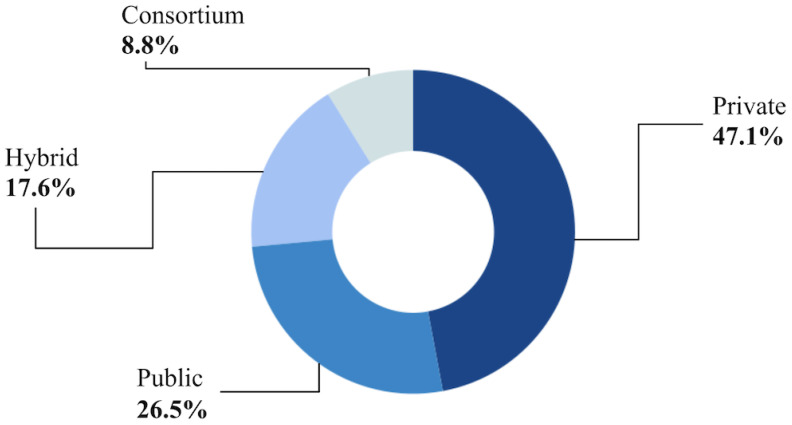
Permissioning methods used by blockchain networks.

According to the definitions and what was presented, 34 studies directly cited that their networks were configured with one of the permissions methods mentioned; of these, 16 (47.1%) were private networks, 9 (26.5%) public, 3 (8.8%) consortium, and the remaining 6 (17.6%) used more than one of the methods and were classified for this systematic review as being hybrid, as shown in Figure 4.

Another relevant point to be analyzed is the use of metrics to measure the performance of the blockchain implementation. To understand the parameters used to evaluate the performance and quality of the solutions included in this review, we analyzed the metrics most commonly employed in these studies. It was used as a reference the metrics cited in Ebad (2023) [103] and selected the following:

- Access Time.
- Block Transmission Rate/Throughput.
- Mining or Reading Time per Block.
- Block per Second.
- Transaction Latency.
- Transaction Packaging Time.
- Transaction Overhead.
- Transaction Throughput.
- Transaction Confirmation Overhead.
- Hash Rate or Quality.

**Figure 5:**
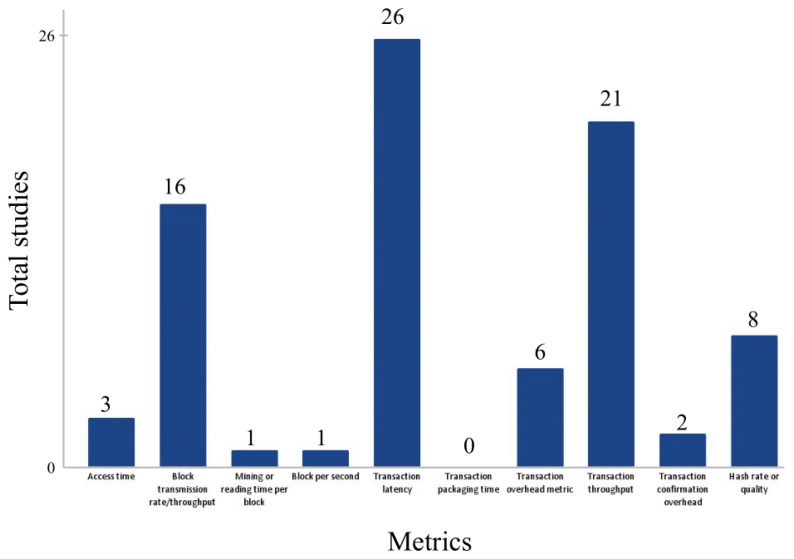
Total studies by metrics mentioned.

As shown in Figure 5, among the most recurring metrics, “Transaction Latency” and “Transaction Throughput” stand out the most, being present in 36% (26 studies) and 29% (21 studies) of the studies, respectively. The concern with latency suggests that response speed is a common priority in blockchain solutions, especially for systems requiring rapid transactions. On the other hand, throughput highlights a concern with transaction processing capacity and scalability. Another frequently used metric is “Block Transmission Rate/Throughput”, present in 22% (16 studies), suggesting that block-level performance is also an important consideration in studies focused on scalability. It was observed that most of the articles chose to evaluate metrics beyond those listed, likely to address the specific needs of their blockchain systems, reflecting flexibility and a focus on the unique requirements of each application. More details about the metrics mentioned for each study can be found in Table S1.

### 3.3. Rq03—How Often Have Articles Related to the Subject Been Published in the Period Analyzed (Distribution by Journal, Geographical Area, and Year)?

**Figure 6:**
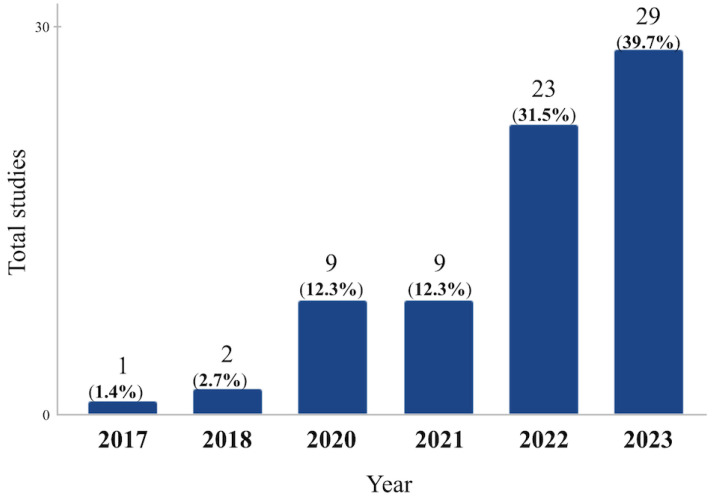
Total publications per year.

To answer RQ03, two factors were taken into account: the number of publications per year related to the subject and the geographical distribution of these publications. With regard to publication by year, the analysis of the primary studies included in this review shows an increase over the years, with most of them published in 2023 with 29 (40%) studies, followed by 23 (31%) publications in 2022, and 9 (12% each) in the years 2021 and 2020 (Figure 6).

**Figure 7:**
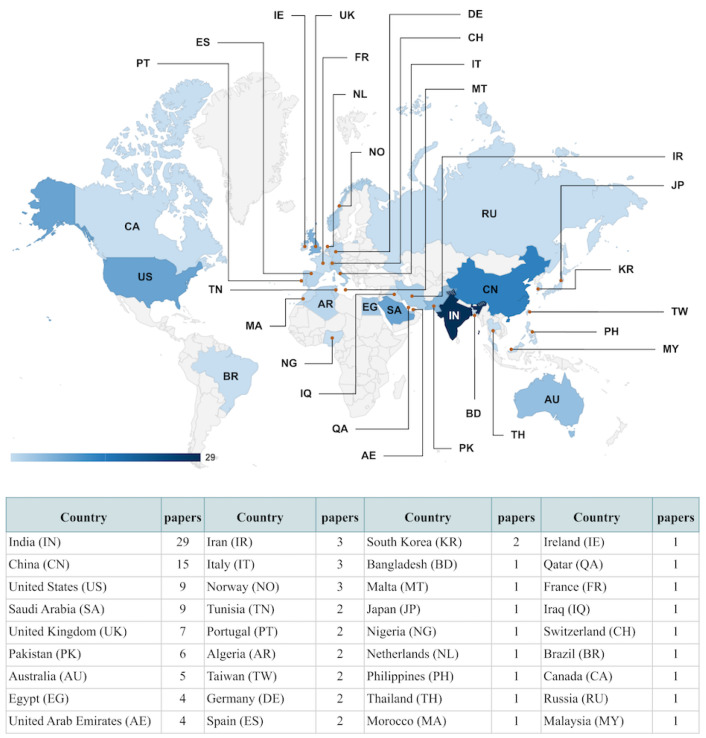
Geographical distribution of the articles analyzed in the systematic review.

In order to visualize the geographical distribution of the articles included in this review, the analysis was carried out considering the countries of each of the authors. Thus, in some cases, an article was developed by researchers from different countries, an aspect that denotes international scientific cooperation on the subject of blockchain. Figure 7 illustrates the distribution of research related to the use of blockchain in health information systems worldwide. In terms of countries, India stands out significantly, as the majority of the articles selected for this review were conducted by institutions located in this country, with a total of 29 articles. China followed with 15 studies, while the United States and Saudi Arabia conducted 9 studies each. The United Kingdom contributed 7 studies and Pakistan 6 studies; more details are available in Figure 7.

### 3.4. Rq04—How Do Blockchain Implementations in Health Information Systems Rank in Terms of Security, Privacy, Data Sharing and Distribution?

In order to classify the primary studies according to the main problem that they wanted to solve with the use of blockchain technology, four groups were identified:

- Security: Studies developed with a focus on solving problems related to data security.
- Privacy: Studies developed with a focus on solving problems related to the privacy of users involved in data transactions.
- Data sharing: Studies developed with a focus on solving problems related to the sharing of information between network participants.
- Distributed data: Studies developed with a focus on solving problems related to the storage and sharing of data among various network participants (or nodes), rather than being centralized in a single location or server.

It is important to note that most of the studies considered more than one of these approaches; for example, they sought to solve problems of privacy and security, data sharing and security, and distributed data and privacy. We therefore sought to select the one that was most relevant to the problem that this work set out to solve. In cases where the focus was on more than one of the approaches and there were insufficient criteria to choose just one of them, the studies were related to more than one, i.e., two approaches were counted.

**Figure 8:**
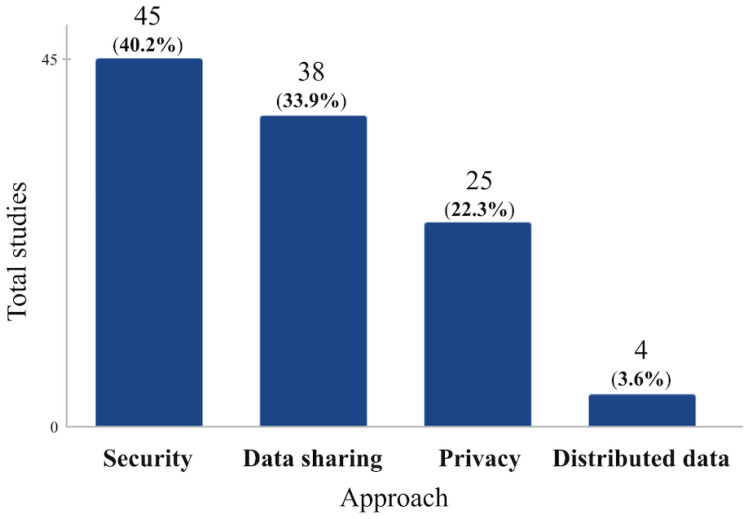
Approaches discussed in the studies analyzed.

According to the pre-established classification, 45 of the studies made use of blockchain technology to solve security problems, another 38 were interested in using it for data sharing purposes, 25 of them made use of the benefits of the technology when it comes to privacy, and finally, 4 of them used decentralization as a means of solving the problem faced, as can be seen in Figure 8.

## 4. Discussion

This systematic review analyzed and organized the use of blockchain technology in health information systems, identifying the main areas of use, the progress made, and the limitations and challenges in this field. One of the findings of this research indicates that blockchain technology has been widely used in various tools in the health context, practically all over the world. The most commonly used practices and models for implementing blockchain in health information systems were the Ethereum and Hyperledger Fabric platforms, with an emphasis on private (47.1%) and public (26.5%) networks. The Ethereum and Hyperledger Fabric platforms are considered emerging technologies, each specialized according to a specific market need. Ethereum has introduced smart contracts, so users of health information systems can make transactions directly, without the need for a centralized entity. Also in the context of market demands, Hyperledger technology became known because it included a set of privacy requirements for companies, cooperations, and institutions. These main characteristics stand out and help justify why both blockchain technologies have been the most cited in research involving health information systems.

The analysis of publication frequency showed a steady increase during the period analyzed, with diverse geographical distribution and a variety of scientific journals. Considering the classification, blockchain implementations in healthcare systems are mostly applied to solve security problems (45 studies), data sharing (38 studies), privacy (25 studies), and decentralization (4 studies), which indicates a greater emphasis on the protection and integrity of patient data. This is a relevant issue, especially when it involves patient data, as integrity, privacy, and confidentiality must be guaranteed. These aspects are mandatory in environments of technological convergence, in which interoperability imposes on health technology ecosystems the need to share data securely [12,104,105,106,107,108]. This is a recurring requirement in the global context of health systems, especially discussed in countries that are implementing the strategic digital transformation agenda recommended by the World Health Organization [109].

The main results reveal a predominance in the use of blockchain technology in electronic health records, covering 63% of the cases studied [11,14,16,17,34,35,36,37,38,39,40,41,42,43,44,45,46,47,48,49,50,51,52,53,54,55,56,57,58,59,60,61,62,63,64,65,66,67,68,69,70,71,72,73,74,75]. This application highlights the search for solutions that guarantee the security and traceability of patient data. In addition, 8.2% of blockchain implementations were identified in the Internet of Medical Things, which highlights the growing integration of this technology with connected medical devices to improve the management and monitoring of patient health data [76,77,78,79,80,82,110]. This is also justified by the imposing dynamics of technological convergence, which is occurring at a very fast pace in the face of the digital transformation of health, which today’s societies are experiencing in almost every part of the world, and which is immersed in the context of the 4th industrial revolution—the trend involving the integration of things, employing interoperable digital platforms, which start to connect and promote interaction online.

As regards the difficulties faced during the implementation of blockchain, only 10 of the articles presented more directly discuss the limitations related to the technology itself and not necessarily directed at the proposed application. Among these challenges, some were more recurrent, such as those shown in Table 6.

To address the storage problem, studies [65,84,111] used an off-chain database, minimizing the storage load on the blockchain. Off-chain transactions are transfers that occur outside the main blockchain network. In this way, the solutions become scalable in the face of a large number of transactions, and off-chain storage does not impact the size of the ledger. In [16], the scalability and performance challenges of the healthcare system are addressed with the implementation of deep learning models. The study applies a distributed approach, dividing the transaction workload and data processing, thereby improving the overall scalability of the system.

To minimize the effect of high transaction fees, a study [90] used the Interplanetary File System (IPFS) along with Hyperledger Fabric, which can handle data at a considerably faster speed than traditional blockchain. Meanwhile, [46] mitigated the issue of high transaction fees simply by switching from the public Ethereum blockchain to the private Hyperledger Fabric blockchain.

The global reach of blockchain technology and the continuous increase in studies in this area indicate that blockchain is becoming a mature and widely accepted technology. This growth reflects confidence in blockchain technology as a secure and transparent solution to various challenges. The geographical diversity of the studies and the cooperation between different countries show a global effort to explore and implement this technology in various sectors, such as health information systems, demonstrating its wide applicability. This movement is heading towards a future in which blockchain will be a central technological infrastructure that can promote innovation, security, and efficiency in a variety of processes.

Public health crisis scenarios, such as the COVID-19 pandemic, have demonstrated the global urgency of inducing and promoting global interoperability of information in the health context [104]. This movement can be seen as the globalization of health, in which data from users of health systems can be shared and analyzed securely by health authorities anywhere in the world, as a way of more effectively preventing and predicting new global public health crises. However, for this to happen, a global effort is needed so that countries can implement public digital health policies that are aligned with the Global Strategy on Digital Health 2020–2025, proposed by the World Health Organization (WHO). Therefore, it is necessary to pay attention to the Global Initiative on Digital Health (GIDH), a network promoted and managed by the WHO, institutions, and government technical agencies which are actively engaged in supporting national digital health transformation [109,112].

The adoption of blockchain technology in healthcare information systems has proven to offer important benefits in terms of privacy, security, data sharing, and interoperability. However, compliance with data protection laws is a significant concern as it impacts patient confidentiality and compliance with regulations such as the LGPD in Brazil [10,113,114], the European Union’s General Data Protection Regulation (GDPR) in Europe, the California Consumer Privacy Act (CCPA) [115] in California, and the Health Insurance Portability and Accountability Act (HIPAA) in the US [95,116]. Given this, blockchain solutions must address principles such as data minimization, integrity, confidentiality, legality, fairness, and transparency [95]. Appropriate measures must be implemented to protect patient identities and ensure compliance with data protection regulations [88].

It is also important to consider the use of agile methods in this process of digital transformation in the global healthcare context. Agile methodologies, which emphasize short, iterative development cycles, allow blockchain solutions to be adapted quickly to the changing needs of the healthcare sector [104,117]. The transparency and traceability inherent in blockchain, when combined with the flexibility and speed of agile methodologies, create an environment conducive to innovation. Thus, the integration of these technologies can transform digital health, providing more robust, reliable, and patient-centered systems.

## 5. Conclusions

This article revealed that blockchain technology has significant potential to transform health information systems, providing robust solutions to the challenges of security, privacy, and interoperability. The studies analyzed have shown that the application of blockchain can improve reliability and efficiency in health data management, from electronic records to data authentication and secure information sharing. Geographical diversity and cooperation between countries show a global movement towards the adoption of this technology, which reinforces its maturity and growing acceptance in the health sector.

However, despite the promising advantages, the implementation of blockchain in healthcare systems still faces significant challenges, such as the need for standardization, regulatory issues, and integration with existing infrastructures. In addition, the analysis identified areas that require further research, especially with regard to the economic impact and scalability of blockchain-based solutions. Even with the high rate of adoption and implementation, it is essential to create a favorable environment for blockchain adoption in order to drive innovation and ensure that solutions using blockchain are implemented effectively and securely.

Therefore, to maximize the benefits of this technology, it is essential that future research addresses these gaps and that there is ongoing collaboration between researchers and health professionals. Global interest can make their development collaborative and continuous, turning challenges into opportunities for significant progress in the quality of public health services.

## Figures and Tables

**Table 1:** Research questions.

RQ	Description
01	In the current scenario, considering the context of health information systems applications, in which tools has blockchain technology been used?
02	What are the most used practices and models for implementing blockchain in health information systems?
03	How frequently have articles related to the subject been published in the period analyzed (what is the distribution by magazine, geographic area, and year of publication)?
04	How do blockchain implementations in healthcare information systems rank in terms of security, privacy, data sharing, and distribution?

**Table 2:** Inclusion Criteria.

IC	Description
01	Articles published from 2012 to 2023
02	Articles published in journals
03	Articles in the fields of technology, engineering and/or computer science
04	Articles in English or Portuguese

**Table 3:** Exclusion Criteria.

EC	Description
01	Duplicate articles
02	Other review articles
03	Studies that do not fit the scope of the research object
04	Articles that do not allow or provide access to the full text

**Table 4:** Quality Assessment Criteria.

QA	Description
01	Are the benefits of using blockchain discussed?
02	Is there a detailed description of the tools and technologies used?
03	Is the problem well defined?
04	Is there a practical application of the developed solution?

**Table 5:** Relationship between the models used and the distribution of blockchain implementation in health information systems.

Model	Application	Total
Ethereum	Electronic health record	18
	Data sharing (COVID-19)	3
	Vaccination card (COVID-19)	3
	Medical data search	1
	Non-fungible tokens	1
	Internet of Medical Things	1
	Data link	1
	Authentication for health	1
	COVID-19 vaccination	1
	Medical cyber–physical systems	1
	Contact tracking	1
	Medicine traceability	1
Hyperledger	Electronic health record	16
	Internet of Medical Things	3
	Blood product management	1
Ethereum and Hyperledger	Electronic recipe	2
	Vaccination card (COVID-19)	1
Exonum	Electronic health record	1

**Table 6:** Most common limitations between articles.

Challenge	References	Details
Scalability	[16,46,54,72,87,88,111]	Blockchain transaction times are often long, which in turn affects the size of the blockchain
		The use of blockchain technology can cause a delay in processing the data generated, as it is more difficult to scale up due to its consensus method
Transaction rate	[16,46,54,90]	There are limitations on the number of transactions that can be processed per second. This can result in transaction delays and network congestion when there is high demand
		Blockchain platforms process only a few transactions per second, which becomes problematic as millions of transactions need to be processed in real time
		In addition, the concept in blockchain that a node needs to pay some fees for the transaction is also a notable disadvantage
Storage capacity	[65,84,88]	Health data can be vast and require significant storage capacity. Blockchain technology inherently stores all transaction data on all nodes, which can lead to substantial storage requirements
		The storage capacity of the blockchain is quite limited, so the introduction of voluminous contact data would greatly affect the efficiency of the blockchain. For example, the maximum block size limit in Bitcoin is 1 MB
		The inclusion of medical images/documents in the block is not possible, as it has limited capacity. However, large medical images/documents are always part of the data in an e-health system

## Data Availability

Not applicable.

## Supplementary Materials

The following supporting information can be downloaded at https://www.mdpi.com/article/10.3390/ijerph21111512/s1. Table S1: Summaries of the articles analyzed in the review.
