# Supplementary material for: Blockchain in Health Information Systems: A Systematic Review
Source: Int J Environ Res Public Health. 2024 Nov 14;21(11):1512. doi: 10.3390/ijerph21111512 (PMC11593537; doi:10.3390/ijerph21111512)
Supplement: Supplementary file 1 [file ijerph-21-01512-s001.zip › ijerph-3211785-supplementary.pdf]

Supplementary Material

Blockchain in Health Information Systems: A Systematic Review

Authors: Aleika Lwiza Alves Fonsêca*, Ingridy Marina Pierre Barbalho, Felipe Ricardo dos Santos Fernandes, Ernano Arrais Júnior, Danilo Alves Pinto Nagem, Pablo Holanda Cardoso, Nícolas Vinícius Rodrigues Veras, Fernando Lucas de Oliveira Farias, Ana Raquel Lindquist, João Paulo Q. dos Santos, Antonio Higor Freire de Morais, Jorge Henriques, Marcia Lucena, Ricardo Alexsandro de Medeiros Valentim

***Correspondence:** aleika.alves@lais.huol.ufrn.br

**Article name**	**Reference**	**Objective**	**Problematic**	**Metrics evaluated**
Blockchain-Based Privacy Preservation for IoT-Enabled Healthcare System	Sharma et al. (2023) [94]	Develop and evaluate decentralized information systems that guarantee user privacy, allowing the secure exchange of confidential information and protecting against unauthorized use of data	Paper-based medical prescriptions have several drawbacks (because they are paper, they are slow to process and susceptible to manipulation, unauthorized reproduction and errors).	Block transmission rate/throughput<br>Transaction latency<br>Transaction throughput
Harmonizing Sensitive Data Exchange and Double-Spending Prevention Through Blockchain and Digital Wallets: The Case of E-Prescription Management	Schlatt et al. (2023) [95]	Study the case of electronic prescriptions and develop a decentralized system for managing these prescriptions using blockchain technology and SSI digital wallets	Current implementations of electronic revenue management systems create centralized data silos, leaving user data vulnerable to cybersecurity incidents and preventing interoperability. Recent literature uses blockchain infrastructure for this, but problems remain	Block transmission rate/throughput<br>Transaction latency<br>Transaction throughput
A blockchain-based protocol for tracking user access to shared medical imaging	de Aguiar et al. (2022) [34]	Establish a protocol for sharing medical data, including images, based on a blockchain architecture.	Recurring concern about the leakage of confidential and sensitive data	Access time<br>Transaction latency<br>Hash rate or quality
Blockchain-based secure medical data outsourcing	Benil and Jasper (2023) [35]	Develop an authentication and verification scheme to safeguard the network against	The centralization of cloud computing raises challenges for privacy protection and data security	Hash rate or quality

with data deduplication in cloud environment		impersonation, falsification, and modification attacks.		
Cloud-based security on outsourcing using blockchain in E-health systems	Benil and Jasper. (2020) [36]	Implement a blockchain-based cloud data storage system to guarantee the audit, integrity and secure tracking of medical data, allowing the visualization of change records	The lack of confidentiality and integrity factors leads to various security problems in sensitive health information	Hash rate or quality
BCSoM: Blockchain-based certificateless aggregate signcryption scheme for Internet of Medical Things	Tomar and Tripathi (2023) [76]	Develop a distributed system that ensures data confidentiality and authenticates devices/patients effectively	IoT connected devices have grown a lot using only a centralized server and this represents overload, single point of failure and security attacks.	Transaction latency<br>Transaction throughput
Collusion resistant multi-authority access control scheme with privacy protection for personal health records	Wu et al. (2023) [37]	Build an access control scheme that protects personal data privacy and allows malicious users to be tracked and held accountable.	Patient data is stored in the cloud and can suffer from unauthorized access and disclosure of private data	
Scalable blockchain model using off-chain IPFS storage for healthcare data security and privacy	Jayabalan and ; Jeyanth (2022) [38]	Integrate blockchain with IPFS to improve privacy and security in the healthcare system, making it more secure, scalable and robust.	Hospitals do not have an effective and secure data-sharing mechanism, which leads to monetary and resource losses if a person visits different hospitals.	
Blockchain-based electronic healthcare record system for healthcare 4.0 applications	Tanwar, et al (2020) [39]	Propose a solution for access control with symmetric key encryption for a different healthcare provider and implement a permission-based EHR sharing system	With the growth of health information being saved on the internet, there is concern about misleading information and violation of patient privacy	Block transmission rate/throughput<br>Transaction throughput
Improving security of medical big data by using Blockchain technology	Sharma et al. (2021) [40]	Develop a smart contract-based architecture to improve the security and privacy of large volumes of data in healthcare.	In the modern healthcare system, big medical data faces many security problems due to the presence of hackers and malicious users	Access time<br>Block transmission rate/throughput<br>Block per second<br>Transaction latency<br>Transaction throughput

Cooperative Blood Inventory Ledger (CoBIL): A decentralized decision-making framework for improving blood product management	Bhandawat et al. (2022) [110]	Create a system based on blockchain and smart contracts to promote cooperation at all levels of the blood supply chain, while maintaining privacy and competition between entities.	e-Health systems raise several issues involving security, privacy, scalability, computational complexity, cost, inconsistent patient profiles, a central point of failure, and much more	
MedRSS: A blockchain-based scheme for secure storage and sharing of medical records	Sun et al. (2023) [41]	Develop a blockchain-based consensus-sharing solution for medical record keeping.	Medical data is increasing dramatically. Centralized systems suffer from privacy violations, data manipulation, and insufficient sharing	Block transmission rate/throughput<br>Transaction throughput
Blockchain-based efficient tamper-proof EHR storage for decentralized cloud-assisted storage	Ramesh et al. (2023) [42]	Create an efficient blockchain-based tamper-proof model for storing electronic health records (EHR) in the decentralized Interplanetary File System (IPFS) in the cloud.	Patients have no direct control of their electronic records while organizations can access outsourced EHRs when necessary. This can cause security problems.	Block transmission rate/throughput<br>Transaction latency<br>Transaction overhead<br>Transaction throughput<br>Transaction confirmation overhead
A blockchain-based keyword search scheme with dual authorization for electronic health record sharing	Yang et al. (2022) [43]	Propose a new blockchain-based, dual-authorization keyword search scheme for sharing electronic health records.	Current electronic health record-sharing schemes have problems with data leakage, incomplete access authorization mechanisms, inflexible operation, low search efficiency, and unreliable cloud server provider	
Blockchain-based COVID vaccination registration and monitoring	Nabil et al. (2022) [83]	Develop a blockchain-based system for recording and monitoring vaccination against COVID-19, aiming to improve the efficiency, transparency, and security of the large-scale vaccination registration process.	Challenges faced by health systems during mass vaccination campaigns, especially in pandemic contexts such as COVID-19 (data security, need for quick access and trust in data)	
A real-time secured medical management system based on blockchain and internet of things	Tiwari et al. (2023) [44]	Create an IoT-based prototype that uses Blockchain technology to eliminate anonymous access to data, ensuring the privacy of patient data through this system.	IoT is subject to privacy and security breaches. The data saved in the database can be breached and this cannot be easily identified	
NFT-IoT Pharma Chain : IoT Drug traceability system	Turki et al. (2023) [81]	Develop a medicine traceability system using blockchain technology and	Lack of transparency, security and reliability in the medicines supply chain.	

based on Blockchain and Non Fungible Tokens (NFTs)		Non-Fungible Tokens (NFTs), integrated with the Internet of Things (IoT) concept, to protect against counterfeit medicines		
A secure blockchain-based e-health records storage and sharing scheme	Shamshad et al (2020) [45]	Use blockchain technology to share electronic records, ensuring the preservation of data privacy and security.	It is a considerable security concern to preserve data confidentiality and privacy in cloud-based eHealth environments	
Hyperledger fabric platform for healthcare trust relations-Proof-of-Concept	Nedaković et al. (2023) [46]	Develop a platform that allows patients to verify the credibility of medical certificates, experiences, and skills.	The relationship of trust between patients, doctors, and health institutions is a challenge	Block transmission rate/throughput<br>Transaction latency<br>Transaction throughput
Efficient Contact Tracing for pandemics using blockchain	Bari et al. (2021) [84]	Minimize the impact of the pandemic by implementing a solution that combines blockchain with a contact tracing application, ensuring user privacy and preventing misuse of data.	Contact tracing systems face some challenges related to issues of medical privacy, data security, and transparency	Block transmission rate/throughput<br>Transaction latency<br>Transaction throughput
An encrypted medical blockchain data search method with access control mechanism	Gan et al. (2023) [96]	Develop an encrypted method for searching medical data on the blockchain, incorporating an access control mechanism to solve problems of single search, low search efficiency, and privacy leakage.	With the growth of electronic medical data, finding this data on the blockchain is a challenge	
Decentralized authentication and access control protocol for blockchain-based e-health systems	Xiang et al. (2022) [47]	Develop a decentralized blockchain-based authentication and access control protocol for electronic health records (EHR) that meets the privacy and security needs of medical data.	Sharing sensitive information in the IoT environment can result in several serious security and privacy problems	
Privacy Preserving Biometric Authentication on the blockchain for smart healthcare	Sarier (2022) [97]	Develop a privacy-preserving biometric authentication system using blockchain technology for application in smart healthcare.	Need for secure authentication and privacy preservation in smart health systems using biometric data	
Self-sovereign identity empowered non-fungible	Zhuang et al. (2023) [98]	Implement a blockchain-based non-fungible patient tokenization system (NFTs),	Health data breaches are on the rise with the exposure of patient records. Data tokenization is an alternative to	Block transmission rate/throughput

patient tokenization for health information exchange using blockchain technology		empowering patients with self-sovereign identity for secure and controlled exchange of health information.	preserve patient data, but it does not adequately address security concerns.	Transaction latency<br>Transaction throughput
The case of HyperLedger Fabric as a blockchain solution for healthcare applications	Antwi et al. (2021) [48]	Conduct an investigative analysis of blockchain technology to determine its suitability for the healthcare industry.	Concern about insecurity due to attacks on medical data and patient records	
Blockchain based context-aware CP-ABE schema for Internet of Medical Things security	Annane et al. (2022) [49]	Propose a solution for the security of decentralized access to health data, protecting patient data against the most common attacks in eHealth applications.	Data from patients with coronavirus increasing significantly. Need for remote monitoring and rapid detection, taking into account data security and accuracy	
Secure and private data sharing in CPS e-health systems based on CB-SMO techniques	Hemalatha et al. (2023) [50]	Develop a secure and private system for sharing data in healthcare systems based on Electronic Health Systems (e-health) and Cyber-Physical Systems (CPS).	The need to guarantee the security and privacy of health data in cyber-physical systems (e-health CPS)	
COUNT: Blockchain framework for resource accountability in e-healthcare	Kumar, et al. (2023) [77]	Develop a blockchain architecture to promote accountability and transparency in medical e-governance.	The outbreak of the COVID-19 pandemic has revealed the gaps in existing medical systems, such as poor management of medical resources and the unavailability of basic requirements for patients	Transaction latency<br>Transaction throughput
Blockchain-based privacy preserving e-health system for healthcare data in cloud	Zhang et al. (2022) [51]	Develop a privacy-preserving blockchain-based e-health system to ensure the security of patients' electronic health records (EHRs).	Malicious doctors can conspire with the cloud storage service provider (CSP) to tamper with patients' EHRs or directly disclose EHR content to other adversaries for profit	Transaction latency
Blockchain Native Data Linkage	Cunningham et al. (2021) [99]	Develop a reference architecture for a data linkage model that replaces the need for a centralized trusted third party with a blockchain-mediated trustless system.	Data providers holding sensitive medical data often need to exchange patient data. Revealing the superset of identifiers for which a supplier needs information can in itself leak sensitive private data.	
A Blockchain-Based Distributed Authentication System for Healthcare	Panda et al. (2021) [100]	Introduce a new blockchain-based decentralized authentication system known as the insurance claim blockchain system.	Patient security and privacy in healthcare systems is a growing issue, as multiple entities (patients, hospitals and health insurance providers) exchange health	

		The proposed system aims to guarantee patient privacy and provide a secure exchange of information and authentication of entities.	information with each other to make decisions	
High-Level Design and Rapid Implementation of a Clinical and Non-clinical Blockchain-Based Data Sharing Platform for COVID-19 Containment	Saleh and Shayor (2020) [85]	Design a high-level blockchain-based platform for clinical research and healthcare intervention data collection where users can exercise control over their data	With progress being made through the spread of the coronavirus, it is crucial to push the boundaries of methods for obtaining clinical data using real-time capture facilities	
eHomeCaregiving: A Diabetes Patient-Centered Blockchain Ecosystem for COVID-19 Caregiving	Alsalamah et al. (2021) [86]	Build a patient-centric family care ecosystem that collects all information in a single blockchain-based mobile healthcare app. The goal is to provide each team member with the support they need to support the patient's continuity of care.	In the COVID-19 pandemic, demand for home care has increased, but existing mobile health apps have failed to equip care providers with the right ecosystem for patient-centered information sharing	
Blockchain-assisted authenticated key agreement scheme for IoT-based healthcare system	Tomar et al. (2023) [78]	Propose a new blockchain-based protocol to establish a secure shared session for authenticated devices to prevent unauthorized access.	Wireless communication channels used by IoT devices are vulnerable to security threats such as unauthorized access and denial of service attacks. Existing solutions rely on a single trusted authority, which can lead to latency problems, centralization, and a single point of failure	Block transmission rate/throughput<br>Transaction latency<br>Transaction overhead<br>Transaction throughput<br>Hash rate or quality
Blockchain-Based E-Medical Record and Data Security Service Management Based on IoMT Resource	Alsudani et al. (2023) [52]	Develop an infrastructure that allows patients to exchange their electronic medical records (EMRs) with different healthcare organizations. The person with full control over the treatment will initiate the EMR transmission.	Patient information is kept in a single hospital and prevents a personalized healthcare system from providing multiple specialists and associated patients with a cohesive, integrated, secure and confidential medical history	
Implementation of Electronic Health Record and Health Insurance	Golda Careline S and T. Godhavari (2022) [14]	Develop a Blockchain-based Electronic Health Records (EHR) and Health Insurance Management System Using Ethereum	Current electronic health record systems are lagging with difficulties such as interoperability and security	

Management System using Blockchain Technology				
Toward the InterPlanetary Health Layer for the Internet of Medical Things With Distributed Ledgers and Storages	Bigini and Lattanzi (2022) [79]	Introduce a new distributed data access layer, the InterPlanetary Health Layer, useful for the healthcare sector to enable data sharing. The implementation of this layer will be based on a DLT network built with the IBM Hyperledger experimental framework.	Medical devices are generally difficult to implement due to privacy regulations and usually use a centralized third party. Enabling data sharing would improve new medical studies	Transaction throughput
A novel blockchain-based electronic health record automation system for healthcare	Chelladurai and Pandian (2022) [53]	Develop a system that represents the healthcare blockchain, capable of creating, updating, and sharing complete patient records on a secure blockchain network.	The number of electronic health records is growing and the accessibility of data, which is fragmented across different providers, is a challenge	Block transmission rate/throughput<br>Transaction latency<br>Transaction throughput
Blockchain for COVID-19: Review, Opportunities and a Trusted Tracking System	Marbouh et al. (2020) [87]	Review several use cases of blockchain technology for COVID-19 and develop a reliable data tracking system based on blockchain.	The COVID-19 pandemic has exposed the health system's limitations, such as the need to validate and verify a large volume of data continuously and quickly	
PRISED tangle: a privacy-aware framework for smart healthcare data sharing using IOTA tangle	Abdullah et al. (2022) [54]	Provide a decentralized structure that allows health data to be shared and transported in a secure and private environment	The emergence of the Industrial Internet of Things (IIoT) has further evolved e-health and with it comes concerns about the security and privacy of health data	Transaction latency
Secure Tamper-Resistant Electronic Health Record Transaction in Cloud System Via Blockchain	Lavanya and Kavitha (2022) [55]	Design efficient and tamper-resistant health data to validate the EHR transaction on the blockchain and ensure that health data cannot be modified by any adversaries	Health records require appropriate technologies to store and share valuable health data	Transaction latency<br>Transaction overhead
MedSBA: a novel and secure scheme to share medical data based on blockchain technology and attribute-based encryption	Pournaghi et al. (2020) [56]	Provide an architecture for sharing and storing medical data integrating attribute-based encryption and blockchain technology	The importance of medical data as an asset for people and the system leads us to be concerned about its security, privacy, and accessibility	
A cognitive approach for	Panwar and	Propose a blockchain-based hash signature	The health sector generates a large amount of data that is	

blockchain-based cryptographic curve hash signature (BC-CCHS) technique to secure healthcare data in Data Lake	Bhatnagar (2021) [17]	technique to protect patients' medical records and share personal data securely	subject to breach and therefore it is expected that the interaction or transaction between the sources that operate the data is reliable and secure	
Patient-centric pre-transaction signature verification assisted smart contract-based blockchain for electronic healthcare records	Chandini and Basarkod (2023) [11]	Develop a patient-centric blockchain assisted by pre-transaction signature verification	Ensuring data security in uncertain channels remains a challenge.	Transaction latency<br>Hash rate or quality
A novel framework paradigm for EMR management cloud system authentication using blockchain security network	Thilagavathy et al. (2023) [57]	Introduce a new framework using a blockchain-based electronic medical records management cloud system that ensures a high-security network.	Cloud-based storage of electronic health data always poses a security risk	
Blockchain technology and IoT-edge framework for sharing healthcare services	ElRahman and Alluhaidan (2021) [58]	Present an IoT-Edge framework for seamless data exchange using data processing and blockchain techniques	Data integrity is difficult to guarantee since data generated from IoT devices is split into parts and stored on several edge servers in various locations	Block transmission rate/throughput<br>Transaction latency<br>Transaction throughput<br>Hash rate or quality
Blockchain-based medical health record access control scheme with efficient protection mechanism and patient control	Yuan et al. (2022) [59]	Propose a blockchain-based sharing and protection scheme	The patient's medical records contain the patient's privacy and should be shared under the control of the patients, not the hospital where this data is acquired	
Secure Electronic Health Record Storage and Retrieval Using Blockchain and Encryption for	Saif et al. (2023) [60]	Present a blockchain-based model to solve the problem and challenges of protecting health records efficiently and securely	Records are subject to cyber-attacks and hackers can obtain the most confidential information about the patient	Transaction latency

Healthcare Application				
Global data sharing of SARS-CoV-2 based on blockchain	Sajedi and Mohammadipanah (2024) [88]	Propose and develop a global data-sharing system on SARS-CoV-2 based on blockchain technology	There is an urgent need to share data on SARS-CoV-2 efficiently and safely on a global scale.	
A lightweight blockchain-based framework for medical cyber-physical system	Kumar and Chatterjee (2022) [101]	Design a blockchain-based framework that will build a trusted network to share information using insecure channels	Various solutions for security are discussed, but most are not viable for the healthcare system in terms of automation, transparency, latency, throughput, security, data tampering, and distribution	Block transmission rate/throughput<br>Transaction latency<br>Transaction throughput
MediBlocks: secure exchanging of electronic health records (EHRs) using a trust-based blockchain network with privacy concerns	Saif et al. (2022) [60]	Develop a secure architecture for exchanging health information using a permissioned blockchain network that will store decentralized electronic health records	Preserving the privacy of personal data. Proporcionar acesso aberto a dados de saúde sensíveis e um intercâmbio bem sucedido de dados de saúde (Interoperabilidade)	Transaction latency<br>Transaction throughput
Blockchain-based end-to-end privacy-preserving scheme for IoT-based healthcare systems	Nasr et al. (2023) [62]	Develop a comprehensive method to protect end-to-end patient privacy and anonymity in an IoT-based healthcare system	A privacy-preserving scheme to protect the privacy of patient data and location is a challenging issue in IoT-based healthcare systems	Transaction latency<br>Transaction overhead
MediChain: Medical data fusion using blockchain-integrated elastic storage	Karmakar et al. (2023) [80]	Store medical data securely and efficiently.	The secure and adaptable storage of critical health information is crucial to the successful operation of the IoMT architecture	Block transmission rate/throughput<br>Mining or reading time per block<br>Transaction latency<br>Transaction throughput<br>Hash rate or quality
Agent-based blockchain model for robust authentication and authorization in IoT-based healthcare systems	Idrissi and Palmieri (2023) [63]	Develop an agent-based and blockchain model for robust authentication and authorization in IoT-based healthcare systems	Patient and professional data are continually subject to serious security attacks	Block transmission rate/throughput<br>Transaction latency<br>Transaction overhead<br>Transaction throughput
Blockchain-enabled healthcare monitoring	Gupta et al. (2023) [64]	Design a Secure Blockchain-Enabled Health Monitoring System Using Transfer Learning	Sharing critical health information securely with various stakeholders such as patients, doctors, and other health	

system for early Monkeypox detection			professionals remains a research challenge	
DAAC: Digital Asset Access Control in a Unified Blockchain-Based E-Health System	Biswas et al. (2022) [65]	Enable cross-communication of medical data between multiple service providers, while keeping individual (technical/administrative) processes and procedures unchanged.	Lack of interoperability and unification of existing independent health service providers. Lack of secure access to patients' digital records by different internal and external elements. The volume and scale of data is extremely large, which complicates unified access control mechanisms	Transaction overhead<br>Transaction confirmation overhead
MEdge-Chain: Leveraging Edge Computing and Blockchain for Efficient Medical Data Exchange	Awad Abdellatif, et al. (2021) [82]	Design a secure and decentralized healthcare system that relies on blockchain and edge computing technologies to provide an intelligent and optimized exchange of medical data between diverse entities such as hospitals and health insurance companies	The exchange of large amounts of information between different e-health entities is a challenge in terms of security, privacy, and network loads. Existing solutions suffer from scalability, computational cost, and slow response	Transaction latency
A Patient-Centric Healthcare Framework Reference Architecture for Better Semantic Interoperability Based on Blockchain, Cloud, and IoT	Gohar et al. (2022) [66]	Build a comprehensive, integrated architecture for data sharing across blockchain, cloud, and IoT to improve interoperability.	There are several problems with centralized EHR systems, such as health data breach problems, a single point of failure, privacy problems with personal and confidential information, and interoperability problems between various systems/data sources	
Secure Decentralized Attribute-Based Sharing of Personal Health Records With Blockchain	Zhang et al. (2022) [67]	Develop a distributed personal health record (PHR) sharing scheme based on blockchain and ciphertext policy ABE (CP-ABE), which enables fast and efficient encryption and decryption.	PHRs have been affected by security issues such as the leaking of personal health information, illegal access to patient data and data tampering. Recent developments in security, using blockchain, have only been partially successful in solving these problems	
Anonymity Preserving IoT-Based COVID-19 and Other Infectious Disease Contact Tracing Model	Garg et al. (2020) [93]	Develop a contact tracing system model using IoT and blockchain	There is concern about the misuse of patients' private data. Most digital contact tracing strategies are not scalable or do not preserve patient privacy, nor do they consider moving objects	

Design and implementation of a New Blockchain-based digital health passport: A Moroccan case study.	Ait Bennace et al. (2022) [89]	Integrate a Blockchain-based private digital health passport to ensure high protection of sensitive information, security, and privacy.	The process of verifying the health pass through the QR code does not respect the individual's privacy, allowing anyone to view and consult the personal information of citizens.	
Block-HPCT: Blockchain Enabled Digital Health Passports and Contact Tracing of Infectious Diseases like COVID-19.	Rashid et al. (2022) [90]	Develop a decentralized blockchain-based digital health passport system with vaccination certificates where contact tracing capabilities are also accommodated	With the rapid growth of COVID-19 cases, a specific strategic response was needed, to guarantee trust, responsibility, and transparency.	
Blockchain in Healthcare: A Decentralized Platform for Digital Health Passport of COVID-19 Based on Vaccination and Immunity Certificates.	Razzaq et al. (2022) [91]	Develop a blockchain-based solution that incorporates consciousness identity, encryption, and decentralized storage through interplanetary file systems.	One of the most difficult aspects of using a centralized storage strategy is maintaining patient privacy and system transparency	
An Architecture and Management Platform for Blockchain-Based Personal Health Record Exchange: Development and Usability Study.	Lee et al. (2020) [68]	Build a blockchain-based architecture for an international health records exchange platform to ensure the confidentiality, integrity, and availability of health records	The security, correctness, and protection of personal health records are essential for medical and health services.	Hash rate or quality
Converging blockchain and next-generation artificial intelligence technologies to decentralize and accelerate biomedical research and healthcare.	Mamoshina et al. (2018) [69]	Assessing the value of time and the combined value of personal data in the context of an AI-mediated healthcare data exchange on the blockchain	One of the main problems for health is the exchange of data and the ability to use data in research and commercial projects. At the same time, the health sector demands a high standard of data privacy	
Decentralized Patient-Centric Report and Medical Image Management System Based on Blockchain Technology and	Mohsan et al. (2022) [70]	Develop a unique proof-of-concept architecture for a patient-centric distributed image management and test reporting system that aims to facilitate patient privacy and control without the	Data exchange is hampered by interoperability issues between different health systems	

the Inter-Planetary File System.		need for a centralized infrastructure.		
Tamper-Resistant Mobile Health Using Blockchain Technology.	Ichikawa et al. (2017) [71]	Develop and evaluate a tamper-proof system using blockchain technology that enables reliable and auditable computing using a decentralized network	Increased use of telemedicine and mobile health, but data tampering is one of the most crucial security risks.	
Blockchain-Powered Healthcare Systems: Enhancing Scalability and Security with Hybrid Deep Learning.	Ali et al. (2023) [16]	Develop a permissions-based blockchain framework for healthcare systems incorporating hybrid deep learning models	Traditional healthcare systems face obstacles in ensuring secure data storage, efficient data sharing, and collaboration between healthcare providers	Access time
NovidChain: Blockchain-based privacy-preserving platform for COVID-19 test/vaccine certificates.	Abid et al. (2022) [92]	Develop a privacy-preserving, Blockchain-based health certificate platform for issuing and verifying COVID-19 test/vaccine certificates.	Different technological solutions are considered, particularly movement documents and traceability applications, but all are vulnerable to fraud and forgery and can influence basic freedoms or be socially unacceptable.	Transaction latency<br>Transaction throughput
A Blockchain Framework to Secure Personal Health Record (PHR) in IBM Cloud-Based Data Lake.	Panwar et al. (2022) [72]	Develop a new framework for managing personal health records (PHR) using IBM's cloud data lake and blockchain platform for an effective health management process.	In the health field, privacy and security are a serious concern for keeping patient data confidential	Block transmission rate/throughput<br>Transaction latency<br>Transaction throughput
Technical Design and Development of A Self-Sovereign Identity Management Platform for Patient-Centric Health Care Using Blockchain Technology.	Harrell et al. (2022) [73]	Describe the technical design decisions of a decentralized patient-centric healthcare identity management system using blockchain technology.	Fragmented health data in different databases. One of the factors that has made it so difficult to integrate clinical data is the lack of a universal identification for everyone	
FHIRChain: Applying Blockchain to Securely and Scalably Share Clinical Data.	Zhang et al. (2018) [74]	Develop a standards-based architecture, integrated with existing health IT systems, to enable secure and scalable sharing of clinical data to improve ongoing,	Many barriers in the technical infrastructure of current health IT systems prevent the secure and scalable sharing of data between institutions	

		collaborative decision support.		
Blockchain-Authenticated Sharing of Genomic and Clinical Outcomes Data of Patients With Cancer: A Prospective Cohort Study.	Glicksberg et al. (2020) [75]	Design, pilot, and launch a decentralized, scalable, efficient, cost-effective, and secure strategy for the dissemination of de-identified clinical and genomic data with a focus on late-stage cancer.	Lack of robust methods for sharing de-identified data	

Supplementary Table S1. Summaries of the articles analyzed in the review.

All references are in the main text references.
